# Facile Ester‐based Phase Change Materials Synthesis for Enhanced Energy Storage Toward Battery Thermal Management

**DOI:** 10.1002/advs.202413703

**Published:** 2025-01-13

**Authors:** Long Geng, Kaifeng Luo, Yixuan Lin, Guo Li, Yitong Cao, Jiateng Zhao, Changhui Liu

**Affiliations:** ^1^ School of Low‐Carbon Energy and Power Engineering China University of Mining and Technology NO. 1 DAXUE ROAD Xuzhou Jiangsu 221116 China

**Keywords:** battery thermal management, covalent modification, phase change energy storage, thermal stability, wide phase change temperature range

## Abstract

With the increasing demand for thermal management, phase change materials (PCMs) have garnered widespread attention due to their unique advantages in energy storage and temperature regulation. However, traditional PCMs present challenges in modification, with commonly used physical methods facing stability and compatibility issues. This study introduces a simple and effective chemical method by synthesizing seven ester‐based PCMs through chemical reactions involving lauric acid (LA) and seven different alcohols. These materials notably broaden the phase change temperature range, exhibiting melting temperature from −8.99 to 46.60 °C, expanding by 203.18% compared to raw alcohol materials. In addition, these samples exhibit excellent thermal stability and high latent heat, with a maximum latent heat value of 182.98 J g^−1^. In subsequent application studies, this material demonstrates outstanding energy storage characteristics and proposed an innovative thermal management method for batteries based on the PCM immersion technique, allowing the battery to maintain a temperature below 60 °C for 20.5 h, while the blank group rapidly reached 60 °C within 0.82 h and increased to 75 °C within 2.35 h. This approach greatly improves temperature regulation, enhances battery safety, and boosts operational efficiency, highlighting the immense potential of the material in advanced energy storage applications.

## Graphical abstract

This study synthesizes seven ester‐based phase change materials (PCMs), significantly broadening their phase change temperature range while exhibiting excellent thermal stability and high latent heat. These properties enhance their application in immersion‐based battery thermal management, allowing for effective temperature regulation, improved battery performance and safety, and optimized heat absorption and release during charge and discharge cycles.

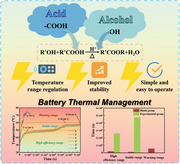

## 1 Introduction

According to related studies, the remaining global natural gas can be used for ≈80 to 100 years, and oil is expected to be depleted ≈2050.^[^ ^1^ ^]^ In addition, the environmental pollution caused by traditional fossil fuels cannot be ignored. Therefore, the development of new energy sources has attracted widespread attention from researchers at home and abroad.^[^ ^2^ ^]^ According to the “World Energy Outlook 2023” report from the International Energy Agency, the global energy system is expected to undergo substantial changes by 2030, with renewable energy accounting for ≈ 50% of the global electricity mix.^[^ ^3^ ^]^ The rapid development of renewable energy application technologies such as wind and solar power has posed a challenge of addressing the intermittency and dispersibility of these renewable energy sources.^[^ ^4^ ^]^ Energy storage technologies, as an important means to ensure the continuity and stability of energy supply, have attracted widespread attention and research worldwide.^[^ ^5^ ^]^ According to the types of stored energy, energy storage technologies include mechanical energy storage, electrical energy storage, electrochemical energy storage, thermal energy storage, and chemical energy storage.^[^ ^6^ ^]^ Among them, thermal energy accounts for more than 70% of global energy consumption and is the primary form of energy for industrial applications and daily life.^[^ ^7^ ^]^

Thermal energy storage can be broadly classified into sensible heat storage and latent heat storage (i.e., phase change energy storage).^[^ ^8^ ^]^ In sensible heat storage, heat is absorbed by changing the temperature of the substance.^[^ ^9^ ^]^ Although sensible heat storage technology is mature and widely used, it has some drawbacks.^[^ ^10^ ^]^ Temperature fluctuations pose challenges to the stability, control performance, and overall efficiency of the system, and may lead to energy losses.^[^ ^11^ ^]^ Additionally, the low heat storage density of sensible heat storage limits its applications in long‐distance and long‐term energy supply.^[^ ^12^ ^]^ However, latent heat storage involves the absorption and release of heat during the phase change process of the substance, while maintaining a constant temperature.^[^ ^13^ ^]^ Therefore, compared to sensible heat storage, phase change storage offers advantages such as higher energy density, greater flexibility, and temperature stability, making it a widely promising energy storage solution.^[^ ^14^ ^]^

Phase change energy storage technology, as an efficient method for thermal energy storage, centers on the selection of PCMs.^[^ ^15^ ^]^ Among various types of PCMs, organic PCMs have attracted attention owing to their tiny supercooling, lower corrosiveness, and stable performance, leading to extensive research and application in relevant fields.^[^ ^16^ ^]^ However, organic PCMs still encounter certain limiting factors in practical applications. One prominent technical challenge is the significant difficulty in regulating the phase change temperature range.^[^ ^17^ ^]^ The phase change temperature range directly affects whether the material can effectively function within a specific temperature range, but the relatively limited selection of organic PCMs currently available restricts their application potential in certain specific temperature ranges.^[^ ^18^ ^]^ Furthermore, while the corrosion and toxicity of most organic PCMs are relatively low, some materials still present potential environmental risks and health issues, which need to be considered and addressed during material development and application.^[^ ^19^ ^]^ Currently, the control of PCM temperature ranges primarily relies on physical methods.^[^ ^20^ ^]^ This is because physical methods can achieve precise control over the phase change temperature without altering the chemical nature of the material, by modifying external conditions.^[^ ^21^ ^]^ For instance, the method of melt blending can prepare eutectic materials by mixing different components in appropriate ratios, so that the phase change temperature reaches the desired temperature range.^[^ ^20a^ ^]^ Additionally, using additives is a common method, wherein introducing specific adjuvants can fine‐tune the phase change behavior of materials, and thus adjust the phase change temperature.^[^ ^21^ ^]^ These physical methods are widely adopted in the research and industrial application of PCMs owing to their ease of operation and suitability for large‐scale use. Xu et al.^[^ ^22^ ^]^ formulated a PCM for cold chain logistics, operational within the 0–8 °C, utilizing an octanoic acid‐lauryl alcohol composite. Liu et al.^[^ ^23^ ^]^ developed a microencapsulated PCM with a core composed of lauric acid (LA) and stearic acid eutectic, and a silica shell, which exhibited suitable melting and freezing temperatures (27.9 and 28.3 °C, respectively), and a high latent heat capacity (with a melting latent heat of 170.3 J g^−1^ and a freezing latent heat of 155.7 J g^−1^). Wen et al.^[^ ^24^ ^]^ developed a ternary eutectic PCM with an adjustable phase change temperature, composed of LA, myristic acid, and palmitic acid, exhibiting suitable thermal properties and good thermal stability. Yang et al.^[^ ^25^ ^]^ developed a novel dodecanoic acid/polyethylene glycol binary eutectic PCM, with a phase change temperature of 22.9 °C and a high latent heat of 173.9 J g^−1^. Zhao et al.^[^ ^26^ ^]^ prepared a binary eutectic PCM consisting of myristic acid and paraffin obtained through a melt‐solution mixing method. It was found that the eutectic PCM exhibited a melting temperature, latent heat, and onset decomposition temperature of 41.99 °C, 171.43 J g^−1^, and 137.86 °C, respectively.

Researchers have made a series of breakthroughs in synthesizing new organic PCMs and precisely controlling their properties. Extensive research has focused on physical methods, successfully optimizing the functionality, and enhancing the performance of PCMs. Although physical approaches undoubtedly offer powerful tools, they are often limited to tweaking existing materials and struggle to fundamentally resolve inherent material flaws.^[^ ^27^ ^]^ In contrast, chemical synthesis plays an indispensable role in materials science, enabling the design and customization of new PCMs with predetermined functions at the molecular level. This method has the potential to address material limitations from the source, leading to more revolutionary improvements in the performance of PCMs.^[^ ^28^ ^]^ However, reports on using chemical reactions to synthesize new PCMs and fully optimize performance through chemical means are still relatively scarce.^[^ ^29^ ^]^

Currently, many PCMs are widely used in research in the field of battery thermal management. Qin et al.^[^ ^30^ ^]^ utilized a composite PCM composed of expanded graphite and paraffin with varying melting points. They established a multi‐parameter electrochemical‐thermal coupling optimization model to optimize the battery spacing, convective heat transfer coefficient, and mass fraction of expanded graphite. Ren et al.^[^ ^31^ ^]^ utilized computational fluid dynamics to investigate the effects of various PCMs on thermal management in lithium‐ion batteries. They found that RT35 and RT50 exhibited optimal performance across different ambient temperatures and that enhancing the thermal conductivity and optimizing the battery pack shape can significantly lower the maximum temperature of the battery. Yin et al.^[^ ^32^ ^]^ conducted experiments to explore the application of PCMs in the thermal management of lithium iron phosphate batteries. They found that gradient‐arranged PCMs can significantly enhance temperature uniformity at high discharge rates, keeping the maximum temperature difference within 3 °C, and proposed an optimal thickness range for the PCMs. He et al.^[^ ^33^ ^]^ proposed an all‐climate battery thermal management structure based on expanded graphite/polymer/paraffin ternary composite PCMs, which can quickly preheat the battery at low temperatures and enhance discharge energy, while effectively dissipating heat in high‐temperature environments.

Therefore, in the research on PCMs for battery thermal management, most efforts primarily rely on simulation methods, with experimental studies mainly focusing on the influence of physical combinations, arrangements, and sizes of simple materials on thermal management performance. However, there is a notable scarcity of studies employing covalent modification techniques to synthesize innovative materials with excellent thermal properties for battery thermal management. Furthermore, the exploration of the potential of material synthesis itself, as well as the thermal management effects in immersion‐type batteries using PCMs, is still relatively limited.

**Figure 1:**
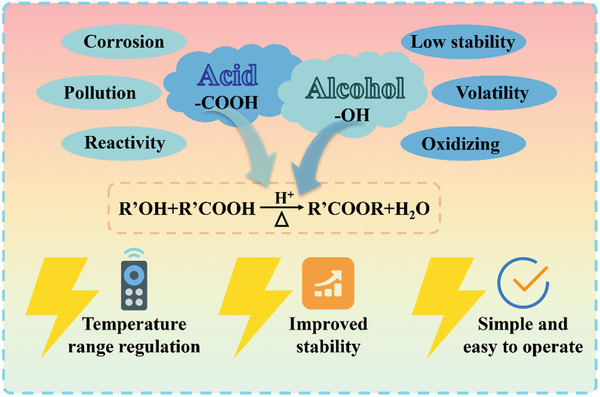
Strategy of this study.

Consequently, this study has successfully developed an ester‐based organic PCM through the classical chemical method of esterification. This research ingeniously selected LA as the reactive acid, combined with seven different alcohols, and through a carefully designed chemical synthesis process, as shown in **Figure** **1**, these newly synthesized esters PCM s successfully addressed many common issues in acid and alcohol‐based materials. Not only did they overcome the corrosion and pollution caused by the reactive sites in the acid and alcohol reactions, but they also effectively circumvented defects such as the ease of oxidation of alcohol‐based raw materials. More notably, these materials have acquired new phase change temperature ranges, bringing additional possibilities to the realms of temperature control and energy storage. Additionally, the ester materials exhibited exceptional thermal stability, providing a solid foundation for their application in various environments. It is worth mentioning that the raw materials used in this study are widely available on the market and are inexpensive, which undoubtedly enhances the appeal of this method. The high efficiency and ease of control inherent to the esterification reaction make this method highly suitable for scaled‐up production, significantly enhancing its industrial application prospects. This study not only contributes significant research findings to the science of PCMs but also presents a novel chemical synthesis pathway, offering valuable insights and inspiration for the design and development of future materials. And, it introduces an innovative battery thermal management method using PCM immersion. This approach greatly improves temperature regulation, enhances battery safety, and boosts operational efficiency, highlighting the immense potential of the material in advanced energy storage applications.

## 2 Results and Discussion

### 2.1 Preparation and Characterization of the Resulting Material

In this study, 7 groups of samples (SP1–SP7) are synthesized using a covalent modification (esterification) strategy. The synthesis methods are detailed in the Supporting Information, and the sample compositions are presented in **Table** **1**. (Among them, the preparation ratios of SP1 and SP5 are from our previous work).^[^ ^34^ ^]^

**Table 1:** Different reaction parameters and the reaction products.

Sample	Raw Materials	Molar Ratio
SP1	LA, Polyethylene glycol 200 (PEG 200)	1: 3
SP2	LA, (Ethanol) EA	1: 1
SP3	LA, (Methanol) MA	1: 1
SP4	LA, PEG 600	1: 1
SP5	LA, PEG 1000	1: 2
SP6	LA, (Hexadecanol) HC	1: 1
SP7	LA, (Ethylene glycol) EG	2: 1

### 2.2 FT‐IR and NMR Analysis of PCMs

**Figure 2:**
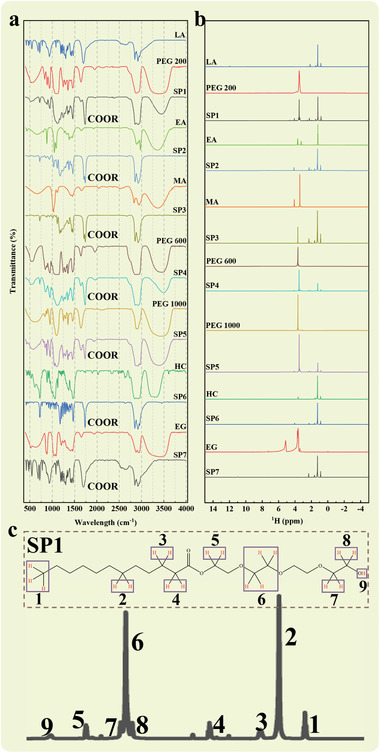
a) FT‐IR and b) 1H NMR spectra of raw materials and as‐samples. c) NMR analysis of SP1.

After establishing the preparation conditions and methodologies, the products are compositionally analyzed to ascertain their exact chemical composition. As depicted in **Figure** **2**a, it is evident that the FT‐IR spectra of all the products diverge from those of the raw materials. Notably, all products display a distinct absorption peak ≈1750 cm^−1^, a feature that is not present in the raw material. This absorption band is associated with the stretching vibration of the COOR group, indicative of the ester molecules present.^[^ ^35^ ^]^ Consequently, it is inferred that the synthesis of the esterified products was successfully accomplished in this study. In addition, 1H NMR analysis revealed that all the samples are single esters, as depicted in Figure 2b. Figure 2c shows the specific peak assignments of the SP1 structure from nuclear magnetic resonance analysis, further confirming the successful synthesis of the product. SP1 and SP5 are synthesized with molar ratios of LA to PEG at 1: 3 and 1: 2. Because it is found that the purity of the sample is not favorable when the molar ratio of the alcohol to the acid is 1: 1. As a result, the quantity of alcohol in this experiment has been moderately increased. Since esterification is a process catalyzed by an acid, with alcohol serving as a nucleophilic agent within it.^[^ ^36^ ^]^ Boosting the quantity of alcohol can speed up the reaction rate and enhance the output of the product. In addition, alcohol can also inhibit the occurrence of side reactions. Besides the formation of an ester through the reaction of alcohol with an anhydride, esterification reactions may also result in hydrolysis, yielding acid and alcohol.^[^ ^37^ ^]^ An excess of alcohol can interact with the produced acid, transforming it into an ester, minimizing side reactions, and thus enhancing the purity of the product. An interesting phenomenon is that when LA reacts with EG at a molar ratio of 2: 1, a diester is expected to form, but this is not the case, and SP7 remains a monoester. This could be attributed to the fact that, following the formation of an ester with the first hydroxyl group of EG, there is an increase in molecular size and steric hindrance, leading to diminished reactivity of the second hydroxyl group.^[^ ^38^ ^]^

### 2.3 Thermal Properties of PCM

After establishing the chemical composition of PCMs, it is essential to examine their thermophysical properties. The phase change temperature and latent heat are two critical parameters for assessing the efficacy of PCMs. These values represent the temperature and energy required to affect a substance's phase change.^[^ ^39^ ^]^ **Table** **2** presents the phase change temperature and latent heat for both raw materials and samples. These samples notably broaden the phase change temperature range, exhibiting melting temperature from −8.99–46.60 °C, expanding by 203.18% compared to raw materials and evenly distributed across various temperature ranges.

**Table 2:** Phase change temperature and latent heat of samples.

Sample	Melting Process		Freezing Process		
	T_m_ (°C)	ΔH_m_ (J/g)		T_f_ (°C)	ΔH_f_ (J/g)
LA	45.96	161.68		38.70	159.72
PEG 600	21.66	115.28		5.95	113.34
PEG1000	36.23	141.12		27.55	133.28
HC	49.02	190.04		45.95	185.46
SP1	−8.99	86.76		−13.00	79.01
SP2	−2.07	91.97		−11.86	96.53
SP3	5.27	104.81		−3.30	107.54
SP4	17.79	85.06		1.56	79.83
SP5	29.35	85.35		2.22	75.57
SP6	38.90	182.98		33.69	192.91
SP7	46.60	109.37		34.26	96.98

^a)^ “T_m_” refers to the melting temperature; “T_f_” refers to the freezing temperature;

^b)^ “ΔH_m_” refers to the latent heat of melting; “ΔH_f_” refers to the latent heat of freezing.

**Figure 3:**
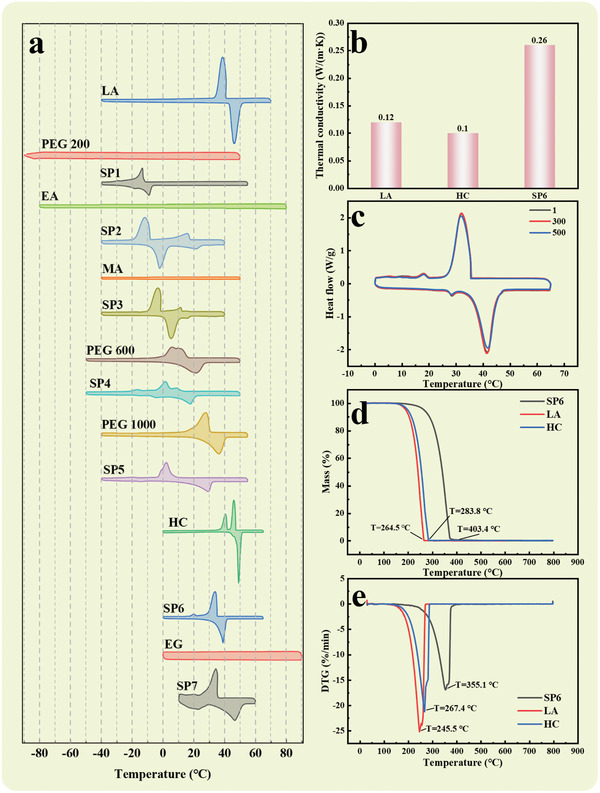
Thermophysical properties and energy storage capacity of the as‐prepared PCM. a) DSC curves of the melting and freezing process of raw materials and samples. b) Thermal conductivity of LA, SP6, and HC. c) Melting and freezing cycling curves of SP6. d) TGA and e) DTG patterns of LA, SP6, and HC (“T” in the figure refers to the temperature).

**Figure** **3** and Table 2 indicate that PEG 200, EA, MA, and EG do not exhibit phase change peaks within the selected temperature range. All these compounds consist of carbon, hydrogen, and oxygen atoms, and their low molecular weights, imply that the van der Waals forces among them are relatively weak, leading them to overcome this force at lower temperatures and induce a phase change.^[^ ^40^ ^]^ Moreover, the molecular structures of these compounds are relatively simple and exhibit symmetry, which can influence how molecules stack and arrange themselves, thus affecting the intermolecular forces present in these compounds. Having a simple molecular structure and symmetry could lead to less robust intermolecular forces. These attributes lead to an extremely low phase change temperature for them; hence, no phase change peaks are detected in the selected temperature range. However, after undergoing esterification reactions with LA, the esterification products SP1, SP2, SP3, and SP7 exhibited distinct phase change peaks within the same temperature range. What's more, the latent heat of melting for these esterification products is promising, with values ranging between 86.76 to 109.37 J g^−1^. This is because the relative molecular weights of PEG 200, EA, MA, and EG are all relatively small. After undergoing an esterification reaction with LA, the molecular weight of the product increases exponentially, resulting in an increase in the phase change temperature. The reason for this is that PEG 200, EA, MA, and EG have relatively low molecular weights when compared to LA. Upon undergoing an esterification reaction, the resulting product has a significantly higher molecular weight, which leads to an elevation in the phase transition temperature. It indicates that the substances which originally did not undergo phase changes within a specific temperature range have been converted into materials with excellent phase change properties within the corresponding temperature range through the esterification reaction. This not only enhances the thermal energy storage capability of the material but also significantly enhances its potential for application in the field of thermal management.

SP4, SP5, and SP6 exhibit varying degrees of melting temperature reduction compared to PEG 600, PEG 1000, and HC, with respective decreases of 17.87%, 18.99%, and 20.64%. Moreover, the latent heat of melting is somewhat weakened, yet it remains within an acceptable range, with values of 85.06, 85.35, and 182.98 J g^−1^, respectively. This is because, during the dehydration esterification reaction between acids and alcohols, the breaking of hydrogen bonds weakens the intermolecular forces, which lowers the phase change temperature of the product.^[^ ^41^ ^]^ Although the esterification reaction in this process helps to increase the molecular weight of the product, considering that PEG 600, PEG 1000, and HC inherently have higher molecular weights compared to LA, means that the overall impact of this increase is relatively minor.

Supercooling is one of the important indicators for evaluating the performance of PCMs. A lower supercooling can facilitate the rapid release of stored thermal energy by the PCM, effectively matching the storage temperature with the heat release temperature, which significantly enhances the efficiency of thermal energy utilization.^[^ ^42^ ^]^ According to the data presented in Table 2, supercooling can be calculated as the difference between the melting temperature and the freezing temperature. In this study, except for the SP5 sample, which exhibited a higher supercooling of 27.13 °C, the other samples demonstrated excellent supercooling performance, with the lowest being only 4.01 °C. This indicates that most samples possess good thermal energy release, contributing to their effectiveness in practical applications.

Furthermore, thermal conductivity is a crucial thermodynamic parameter used to quantify the rate of heat transfer in materials.^[^ ^43^ ^]^ According to the results shown in Figure 3b, the thermal conductivity of SP6 is 0.26 W/(m·K), which represents an increase of 160% compared to HC and 117% compared to LA. It shows that SP6 has a significant improvement in thermal conductivity compared to the raw material. It can improve the thermal response speed of PCMs, achieving more efficient energy storage and release, while reducing temperature gradients, ensuring an even temperature distribution, thereby extending the lifespan and enhancing the system safety of material.

Moreover, thermal cycling leads to a reduced rate of heat storage and release, necessitating a longer duration for the heat charging and discharging process, thereby directly affecting operational efficiency.^[^ ^44^ ^]^ This study examines the stability of SP6 over 500 thermal cycles, with a focus on its phase change temperature, latent heat value, and structural stability. As illustrated in Figure 3c, after 500 thermal cycles, the phase change temperature and latent heat value of SP6 remain largely unchanged, indicating excellent thermal stability. This confirms the reliability and durability of SP6 in practical applications.

High thermal stability plays a crucial role in the lifespan, stability, and safety of PCM. This high thermal stability not only significantly enhances the performance and reliability of PCM in various thermal storage applications but also ensures safety and stability in high‐temperature environments.^[^ ^45^ ^]^ Through TGA testing, the change in sample mass with temperature can be observed. Figure 3d shows the mass change curves of HC, LA, and SP6 below 800 °C. It can be seen that the mass of each sample remains stable below 200 °C. As the temperature rises, the mass of LA decreases significantly first, followed by HC. Further analysis indicates that the main components of SP6 completely decompose above 400 °C, with a complete decomposition temperature ≈42.14% higher than HC and 52.52% higher than LA. For a more detailed analysis of the rate of mass change, refer to the DTG curve in Figure 3e. The peaks in the DTG curve indicate major mass loss at different temperatures. Three distinct peaks are located ≈245.5, 267.4, and 355.1 °C, corresponding to LA, HC, and SP6, respectively. The temperature corresponding to the major mass loss of SP6 is ≈32.80% higher than HC and 44.64% higher than LA. Additionally, the maximum mass loss rate of SP6 is ≈21.66% lower than HC and 33.72% lower than LA. Consequently, the excellent thermal stability of SP6 gives it a distinct advantage in thermal management systems, allowing it to remain stable over a wider temperature range, reducing material degradation and loss, thus enhancing overall system efficiency and reliability.

**Figure 4:**
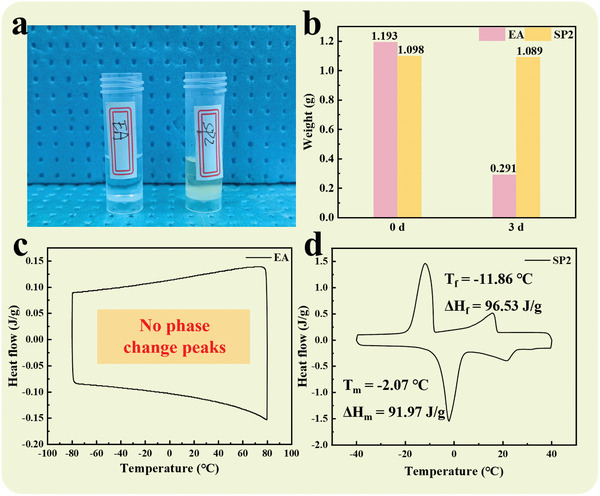
a) EA and SP2 are tested for volatility by exposing them to room temperature. b) Mass before and after three days of EA and SP2. DSC curves for c) EA and d) SP2.

This study investigates the volatility of EA and SP2 at room temperature (≈25 °C). The experiment is designed by placing the two compounds separately in an open environment and observing them continuously for 3 d, as shown in **Figure** **4**a. At the beginning of the experiment, EA has an initial mass of 1.193 g, which decreases to 0.291 g after 3 d, representing a significant loss of 75.61%. In contrast, SP2 has an initial mass of 1.098 g, which decreases to 1.089 g after 3 d, showing a decrease of 0.82%, indicating lower volatility, as shown in Figure 4b. Due to being an alcohol compound with a lower boiling point, EA evaporates quickly at room temperature. In contrast, SP2 is generated by an esterification reaction, exhibiting lower volatility due to its higher molecular weight and more complex molecular structure. Moreover, within the temperature range of −80 to 80 °C, EA does not exhibit a phase change peak, indicating that it cannot be effectively utilized in everyday phase change thermal storage applications. In contrast, SP2 demonstrates favorable phase change behavior, exhibiting significant latent heat of phase change and minimal supercooling. This characteristic enables SP2 to offer better thermal management performance in practical applications. Therefore, the experimental results emphasize the potential application value of esterification reaction in enhancing the thermal stability of compounds and reducing their volatility, especially suitable for industrial scenarios requiring volatility control. This study not only reveals significant differences in the physical properties of the two compounds but also provides valuable data and insights for subsequent research and applications.

### 2.4 Application

**Figure 5:**
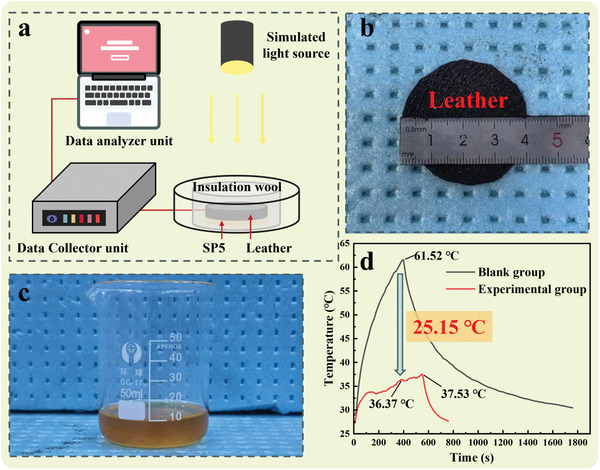
a) Diagram of measurement platform for the photothermal experiment. b) Leather picture and size. c) SP5 physical picture. d) Photothermal experiment test results.

To address the issue of bicycle or electric bike seat overheating due to strong summer sunlight, an experiment was designed to evaluate the effectiveness of PCMs in cooling seat cushions. The PCM chosen, SP5, has a phase change temperature within the range of human thermal comfort. In the experiment, 12.7 g of SP5 (as shown in **Figure** **5**c) is weighed and placed in a beaker, covered with a layer of plastic wrap. The experimental setup includes two groups: a blank group without SP5 and an experimental group with SP5 padded experimental group. For each group, leather samples ≈4 cm in diameter are cut, as shown in Figure 5b, to simulate the outer material of a bicycle seat. These samples are then tested under a simulated sunlight source to mimic real outdoor exposure conditions. The results, displayed in Figure 5d, show that within 7 min, the temperature of the leather samples in the blank group rapidly rose to 61.52 °C, indicating that without any cooling measures, the leather samples absorbed a significant amount of heat in a short time. At this point, the temperature of the leather in the experimental group is 36.37 °C, a reduction of ≈40.88%. Furthermore, the temperature of the leather samples in the experimental group was effectively controlled, stabilizing at no more than 38 °C when SP5 underwent a noticeable phase change. This result demonstrates that SP5 can effectively use its phase change properties to regulate temperature by absorbing and storing excess heat, thus maintaining a lower level of thermal comfort. Additionally, the experiment observed that after removing the light source, the cooling rate of the leather samples in the experimental group was significantly faster than that of the blank group. This phenomenon further verifies the efficiency of SP5 as a PCM in thermal management, providing cooling effects during light exposure and rapidly releasing stored heat to accelerate the cooling process when the light is off. In conclusion, using SP5 as a PCM in bicycle seat cushions can significantly improve thermal comfort during summer rides, which has important implications for enhancing the performance of outdoor sports equipment.

**Figure 6:**
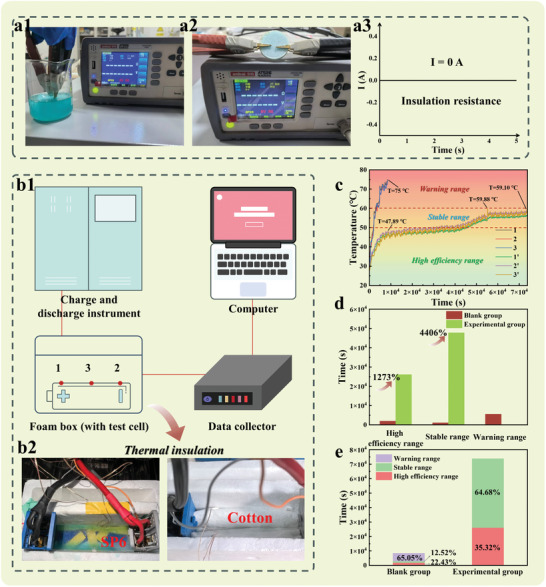
SP6 resistance test in a1) liquid and a2) solid‐state respectively. a3) Current test curve of SP6. b1) Diagram of battery thermal management test platform. b2) Battery insulation thermal treatment. c) Battery surface temperature test curve. d) Comparison of surface temperature maintenance time of two groups of batteries in each temperature range. e) Proportion of each temperature range in two groups of batteries.

The SP6 successfully developed in this study exhibits excellent performance in the field of energy storage, especially showing great potential in battery thermal management. SP6 has a melting temperature of 38.90 °C and a high latent heat of melting of 182.98 J g^−1^. Moreover, it exhibits excellent thermal stability and thermal cycling stability. Therefore, ≈700 g of SP6 is selected for the immersion battery thermal management experiments (Specific experimental steps and detailed parameters of the battery are detailed in the Support Information). First, the resistance of the material is tested using a battery tester, as shown in **Figure** **6**a1 to 6a3, revealing that SP6 is non‐conductive in both solid and liquid states, making it suitable for immersion battery experiments. Subsequently, under the same conditions, this study insulates the batteries with cotton and SP6, respectively, and compares the surface temperatures of the two groups. The results are shown in Figure 6c, where the temperatures of the blank group batteries are labeled as 1, 2, 3, and those of the experimental group as 1′, 2′, 3′. The experimental results indicate that batteries soaked in SP6 can maintain a temperature below 60 °C for up to 20.5 h, whereas the blank group rapidly reached 60 °C in just 0.82 h and continued to rise to 75 °C in 2.35 h, approaching the safety limit of 80 °C. At this point, the surface temperature of the experimental group batteries is ≈27.11 °C lower than that of the blank group, with a reduction rate of ≈36.15%. Furthermore, the rate of temperature increase in the experimental group is significantly slower than that of the blank group. As shown in Figure 6c, this study classifies the operating temperature ranges of the batteries into three levels based on their usage instructions: the high‐efficiency range, the stable range, and the warning range. As shown in Figure 6d,e, SP6 significantly enhances the time the batteries maintain their temperature in the high efficiency and stable ranges, increasing by 1273% and 4406%, respectively, compared to the blank group. Additionally, during the 20.5 h test, the temperature of the batteries in the experimental group completely remains within the high‐efficiency and stable ranges, accounting for 35.32% and 64.68% of the time, respectively. In contrast, the batteries in the blank group spend most of the time in the warning range, accounting for 65.05%. Therefore, SP6 demonstrates exceptional energy storage properties and introduces an innovative approach to battery thermal management using phase‐change material immersion. This method significantly improves temperature regulation, enhances battery safety, and extends operational efficiency, highlighting the strong potential of SP6 for advanced energy storage applications.

**Table** **3** summarizes recent different PCM applied in battery thermal management. It can be observed that recent PCMs for battery thermal management are mostly prepared using simple physical combinations. However, this study fundamentally regulates material properties at the chemical level through covalent modification. In comparison, this material demonstrates exceptional performance across a wide temperature range, effectively meeting the thermal management needs of daily life while offering high latent heat and excellent thermal stability, showcasing strong competitiveness. Furthermore, the material is cost‐effective, nontoxic, and environmentally friendly (see Supporting Information), with simple and effective operation. More importantly, this study not only provides an innovative material synthesis method but also proposes a novel concept of immersive battery thermal management, offering new solutions and technological pathways to address future challenges in battery system thermal management, highlighting significant scientific value and application potential.

**Table 3:** Summaries of different PCM for battery thermal management.

Entry	Materials	T_m_ (°C)	ΔH_m_ (J/g)	Complete Pyrolysis Temperature (°C)
1^[^ ^46^ ^]^	Cross‐linked‐n‐Octadecane@Poly(vinyl alcohol)	32.90 ± 0.20	201.10 ± 2.20	> 100.00
2^[^ ^47^ ^]^	PEG/Polyurethane/Reticulated graphite nanoplatelets	43.80–47.90	113.90–150.40	346.10–360.30
3^[^ ^48^ ^]^	Paraffin/Olefin block copolymer/Expanded graphite	47.42–50.73	176.70–197.70	Step1: 200.00–330.00 Step2: > 400.00
4^[^ ^49^ ^]^	Paraffin/ Graphite powder/ Nickel foam	48.18–50.48	124.53–154.91	∖
5^[^ ^50^ ^]^	Flame‐retardant inorganic composite PCM based on sodium acetate trihydrate	50.20	154.50	245.00
6^[^ ^51^ ^]^	Lauric acid/Expanded graphite@MIL‐101‐NH_2_	45.75	94.55	233.00
This work	Novel ester‐based PCMs based on covalent modification strategies	−8.99–46.60	85.06–182.98	403.40

## 3 Conclusion

This study synthesized a series of new ester‐based PCMs through the esterification reaction of LA with seven different alcohols, aimed at overcoming common limitations of traditional PCMs, such as type restrictions due to temperature control difficulties, performance instability, and compatibility issues. The melting temperature range of the new PCM significantly expanded, achieving a 203.18% increase compared to traditional materials. This extensive temperature range significantly enhances its application potential. Synthetic ester‐based PCMs exhibit outstanding thermal performance, which includes excellent thermal stability and high latent heat, with the highest latent heat of melting recorded at 182.98 J g^−1^. These characteristics render them particularly suitable for energy storage and temperature regulation applications. Moreover, these materials effectively address common problems such as corrosion, instability, and contamination prevalent in acid and alcohol‐based PCMs, while also overcoming the volatility issues associated with some alcohol‐based raw materials. The utilization of widely available and cost‐effective raw materials, combined with the straightforward and scalable process of esterification, underscores the industrial viability of this method. And, it introduces an innovative battery thermal management method using PCM immersion. This approach greatly improves temperature regulation, enhances battery safety, and boosts operational efficiency, highlighting the immense potential of the material in advanced energy storage applications. In conclusion, the development of these ester‐based PCMs not only contributes to the science of energy storage materials but also offers a sustainable, efficient, and innovative solution to the pressing challenges of energy conservation and environmental protection. The findings from this study provide valuable insights and pave the way for future research and development efforts in the PCM industry, promising significant impacts on both technological advancements and practical applications.

## Conflict of Interest

The authors declare no conflict of interest.

## Supporting information



Supporting Information

## Data Availability

The data that support the findings of this study are available from the corresponding author upon reasonable request.
