# Supplementary material for: Facile Ester‐based Phase Change Materials Synthesis for Enhanced Energy Storage Toward Battery Thermal Management
Source: Adv Sci (Weinh). 2025 Jan 13;12(9):2413703. doi: 10.1002/advs.202413703 (PMC11884538; doi:10.1002/advs.202413703)
Supplement: Supplementary file 1 — Supporting Information [file ADVS-12-2413703-s001.docx]

***Facile Ester-based Phase Change Materials Synthesis for Enhanced Energy Storage Towards Battery Thermal Management***

Long Geng, Kaifeng Luo, Yixuan Lin, Guo Li, Yitong Cao, Jiateng Zhao, Changhui Liu*

*School of Low-carbon Energy and Power Engineering, China University of Mining and Technology, Xuzhou, 221116, China. Email: liuch915@cumt.edu.cn*

Contents

[*Experimental* 3](#_Toc184322448)

[*Figure S1* *Preparation process of polyethylene glycol laurate* 3](#_Toc184322449)

[*Table S1 Parameters of battery* 4](#_Toc184322450)

[*Figure S2 Molecular structural formula of SP1* 5](#_Toc184322451)

[*Figure S3 HRMS (ESI) of SP1* 5](#_Toc184322452)

[*Sample Toxicity Testing of SP1* 6](#_Toc184322453)

[*Table S2 Results of toxicity tests* 6](#_Toc184322454)

[*Figure S4 Scale up experiment of SP1 and SP5* 7](#_Toc184322455)

[*Calculation of sample preparation costs* 8](#_Toc184322456)

[*Table S3* *Raw material unit price list form* 8](#_Toc184322457)

[*Figure S5 Cost diagram for SP1~SP7* 8](#_Toc184322458)

# *Experimental*

**Chemicals and materials**

Methanol (MA), ethanol (EA), ethylene glycol (EG), hexadecanol (HC), polyethylene glycol (PEG) and LA are purchased from Shanghai Macklin Biochemical Co., Ltd. Concentrated sulfuric acid (H_2_SO_4_, 95~98 wt%) is supplied by Sinopharm Chemical Reagent Co., Ltd. Ethyl acetate (C_4_H_8_O_2_), sodium bicarbonate (NaHCO_3_), sodium chloride (NaCl), and anhydrous sodium sulfate (Na_2_SO_4_) are purchased from Shanghai Titan Scientific Co., Ltd.

**Chemicals and materials**

Initially, raw materials are combined in a round-bottom flask and subjected to a heat and stir process at 130 °C and 400 rpm for 3 h (For volatile methanol, ethanol and ethylene glycol, the reaction temperature should be gradually increased to a starting temperature of 40 °C), with a small amount of concentrated sulfuric acid introduced dropwise during heating. The reaction yields a product that is then moved to a beaker. Here, ethyl acetate is slowly added to achieve a clear solution. Water is then introduced to the beaker to allow for phase separation. Small increments of NaHCO_3_ are subsequently added until bubbling ceases and the aqueous phase pH nears 7. The solution undergoes multiple extractions in a separatory funnel. After extraction, the oil phase is treated with anhydrous sodium sulfate. The purified product is finally obtained through rotary evaporation at 40 °C under a 0.1 MPa vacuum for 1 h, as shown in Figure S1.


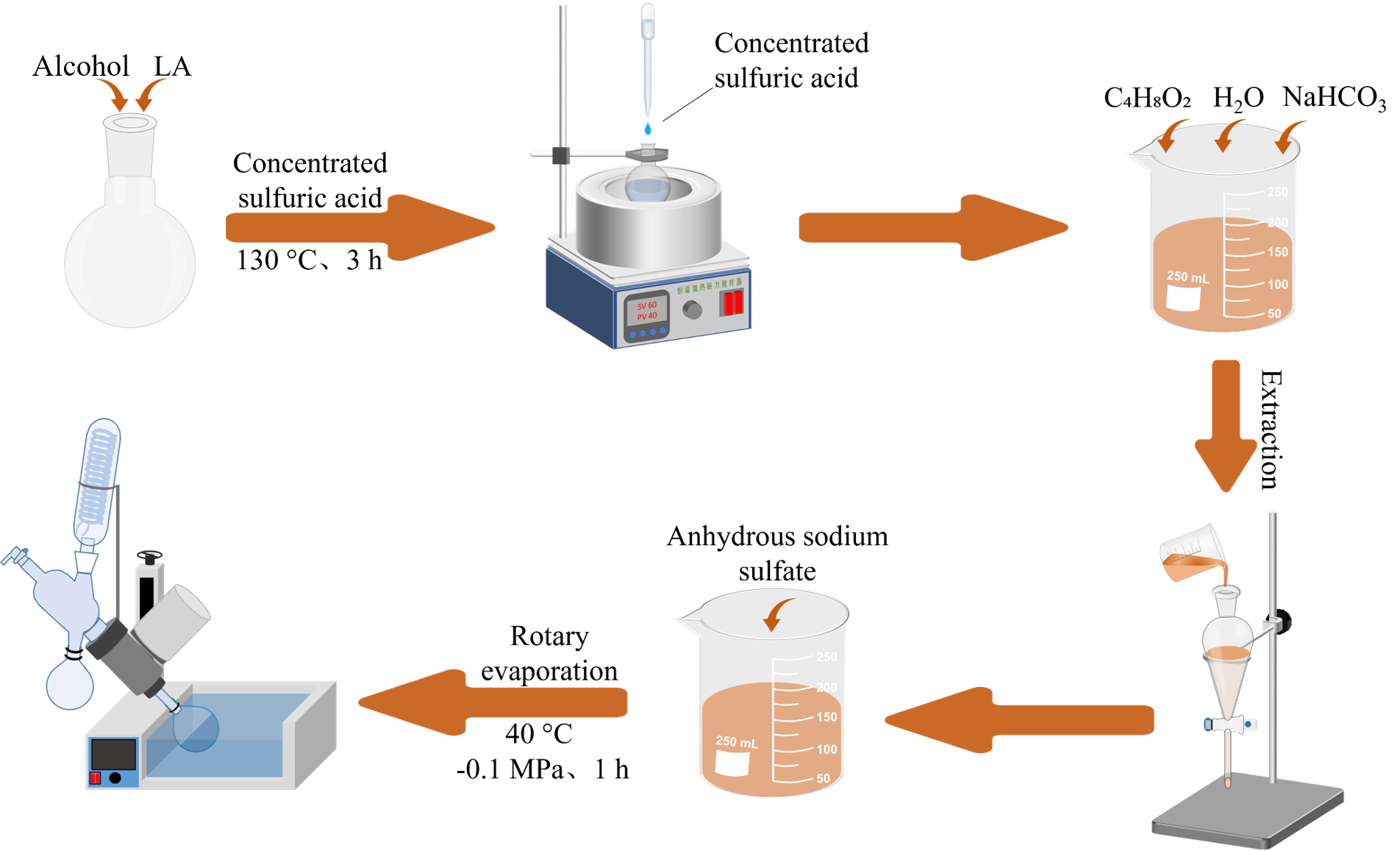


# *Figure S1* *Preparation process of polyethylene glycol laurate*

**Characterization and property measurement**

The composition of the products is examined using a Fourier Transform Infrared Spectroscopy (FT-IR) device, specifically a Brüker VERTEX 70 model. For the investigation of the molecular structures, Nuclear Magnetic Resonance (NMR) spectroscopy is employed, with the collection of ^1^H NMR spectra being performed on a Brüker AV-400 spectrometer. To determine the temperature of phase change, the heat associated with this change, and the stability of the samples across thermal cycles, a Differential Scanning Calorimeter (DSC) from TA Instruments, model DSC 25, is utilized. The thermal conductivity of the samples is measured by employing the transient hot-wire technique, using a Hot Disk 2500-OT thermal conductivity meter, which is produced in Sweden. Thermal stability assessments of the samples are conducted through Thermogravimetric Analysis (TGA) using a TA Instruments SDT Q600 analyzer. This analysis is performed in a nitrogen environment, with the temperature escalating from ambient to 800 °C at a uniform rate of 10 °C per minute.

**Photothermal application experiment**

A photothermal test platform is constructed for this experiment. The test platform comprises a simulated light source with a constant light intensity of 135 mW/cm^2^, a data collector unit, a data analyzer unit, and a testing unit.

**Battery thermal management experiment**

This work uses the battery tester (Anbai, AT526) to measure the resistance of sample SP6 in both liquid and solid states. After confirming that SP6 is non-conductive, immersion thermal management temperature measurement experiments on the battery are initiated using it. The experimental setup, consists of a charge-discharge instrument (NEWARE, CT-4001-60V100A), a computer, a data collector (Agilent, 34970A), thermocouples, lithium iron phosphate battery and a foam box.

High-performance electric loading startup systems and emergency systems impose high demands on lithium-ion batteries as a power source, such as in terms of energy density, high and low-temperature performance, and charge-discharge rate characteristics. Therefore, this work refers to the more mature emergency UPS power supplies and selects a 20 Ah lithium iron phosphate battery for testing and configuration. The detailed parameters are shown in Table S1. The battery thermal management test unit and the thermocouple arrangement on the battery surface described in this work are shown in Fig.6b1, where the battery surface is thermally insulated with cotton/SP6 wrapping; the battery temperature data are measured by three thermocouples located at the positive and negative tabs and the middle part of the battery.

# *Table S1 Parameters of battery*

| 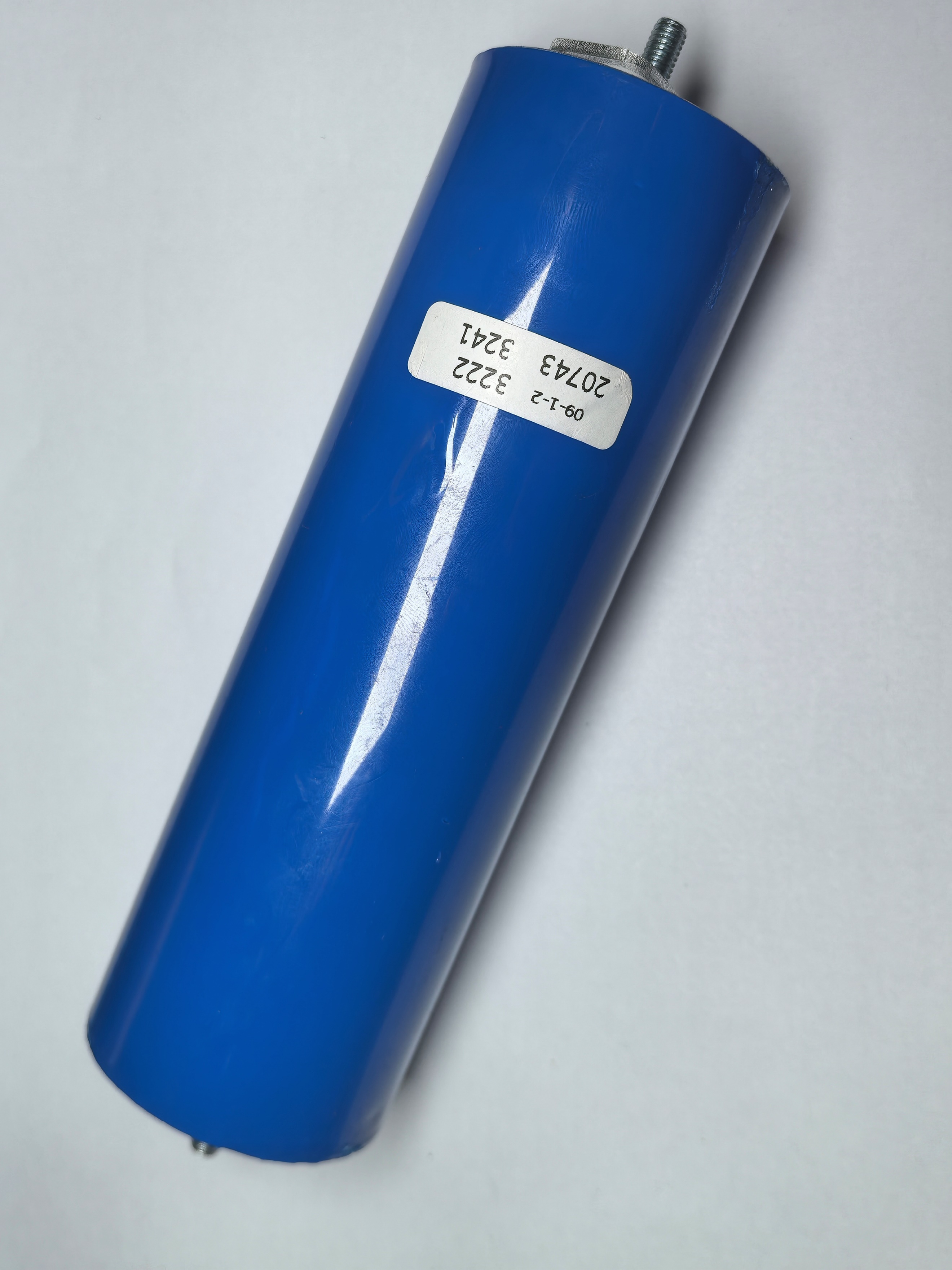  47145 (20 AH) | Category | Parameter |
| --- | --- | --- |
|  | Battery type | LiFePO_4_ |
|  | Battery model | 47145 (20 AH) |
|  | Size | Calibre: 47 mm  Length: 145 mm |
|  | Nominal voltage | 3.2 V |
|  | Discharge rate | 10 C-25 C |
|  | Charging Voltage | 3.65 V |
|  | Discharge termination voltage | 2 V |

The charge-discharge cycle described in this work includes constant current-constant voltage charging and constant current discharging. The specific steps are as follows: First, use a charge-discharge instrument to charge in constant current-constant voltage mode, i.e., charge at 60 A (3 C) until the battery voltage reaches 3.65 V, then switch to constant voltage charging until the current drops to the cutoff current of 1 A (0.05 C). Next, use the charge-discharge instrument to perform constant current discharging at 60 A (3 C) until the voltage drops to 2 V. Finally, repeat the above steps until the test is completed.

HRMS (ESI): calcd for C_20_H_41_O_6_^+^ [M+H] ^+^: 377.2903; found: 377.2892.

# *Figure S2 Molecular structural formula of SP1*

# *Figure S3 HRMS (ESI) of SP1*

# *Sample Toxicity Testing of SP1*

**Experimental materials**

**Sample Information**

Subject: phase change cold storage material; Property: brown liquid; Preparation: weigh 10.0003 g of sample and add evaporated water to 20 mL.

**Experimental animals and feeding environment**

**Animal species:** ICR mice, Grade: SPF grade, number: 20, Sex: half of male and half of female, Weight: 18.07 ~ 19.97 g, Source: Shanghai Shengchang Biotechnology Co., Ltd., Laboratory Animal Production License No.: SCXK (HU) 2021-0002, Quality Certificate No.: 20210002004851

**Feeding environment:** SPF grade animal room, Room number: 348, Temperature of the feeding room is 20 ~ 26 ºC, Relative humidity is 40% ~ 70%, Laboratory Animal Use License No.: SYXK(HU) 2021-0023.

**Feed source:** Shanghai Zhouyu Bio-technology Co., Ltd, Production License No.: Shanghai Feeding Certificate (2021) 04027. Drinking water without restriction.

**Source of bedding:** Shanghai Zhouyu Bio-technology Co., Ltd, Production License No.: Shanghai Feeding Certificate (2021) 04027.

**Instruments:** Electronic balance STX622ZH (WPE-TL0029), Electronic Balance PX224ZH (WPE-TL0241)

**Experimental methods**

**Test basis:** GB 15193.3-2014 "National Food Safety Standard Acute Oral Toxicity Test" (limited method)

**Operating procedures**

- Animals are acclimatized to the experimental animal house environment for 6 d prior to testing.
- Before the test, the animals were divided into 4 cages of 5 animals per cage according to their sex, and were fasted for 4 ~ 6 h and given free access to water.
- The test adopts the limited method, the poison is administered by oral gavage, the volume is 2 mL/100 g, the poison dose is 10.0 g/kg body weight, and the poison is administered once within 24 h. The animals were fasted for 1 ~ 2 h after the administration of the test substance.
- Observe the abnormalities of the animals every day after the poisoning, and perform necropsy on the dead animals during the observation period and the animals executed at the end of the observation period, observe the tissues and organs visually to see if they are abnormal, and perform histopathological examination if necessary. Observation time: 14 d. Weigh the animals at the beginning and end of the experiment and record their weights, and weigh them at least once a week during the observation period.

# *Table S2 Results of toxicity tests*

| Sex | Number of mice | Weight ($\bar{X}$±SD) (g) | | | | Toxicity | Deaths | |
| --- | --- | --- | --- | --- | --- | --- | --- | --- |
|  |  | 0 d | 1 d | 7 d | 14 d |  |  |  |
| **♀** | 10 | 19.10±0.69 | 19.76±0.78 | 25.21±0.91 | 33.28±1.19 | none | | 0 |
| **♂** | 10 | 19.10±0.63 | 19.70±0.60 | 26.32±1.09 | 35.57±1.44 | none | | 0 |

a. Data from received work^[1]^


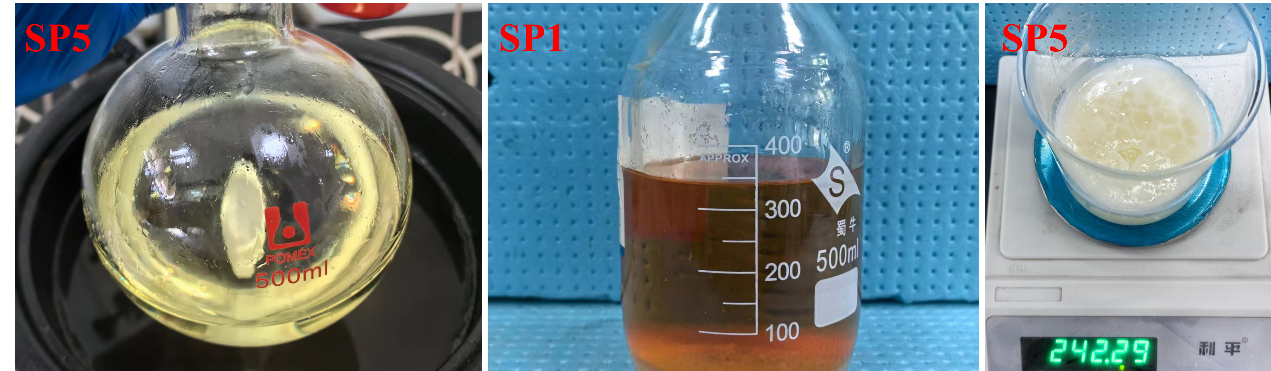


# *Figure S4 Scale up experiment of SP1 and SP5*

# *Calculation of sample preparation costs*

The cost calculation is based on the consumption of 500 g of alcohol (AL) and LA (Laboratory pilot-scale). The unit price per gram of LA and AL is multiplied by the number of grams required, and the cost of the remaining raw materials is added to give the cost of preparing the corresponding esterified product. This is shown in Equation 1. Among these, ethyl acetate can be recycled after spin evaporation.

*C = n_LA_· M_LA_· P_LA_+ n_AL_· M_AL_· P_AL_+ 0.02· (n_LA_+ n_AL_) · M_H2SO4_· P_H2SO4_+ m_C4H8O2_· P_C4H8O2_+ m_NaHCO3_· P _NaHCO3_+ m_NaSO4_· P_NaSO4_ (1)*

Where C indicates the cost, n indicates the number of moles, M indicates the molar mass, and P indicates the unit price per gram, m indicates mass.

# *Table S3* *Raw material unit price list form*

| Raw material | Unit price  (CNY/g) |
| --- | --- |
| PEG 200 | 0.051 |
| EA | 0.060 |
| MA | 0.039 |
| PEG 600 | 0.059 |
| PEG 1000 | 0.079 |
| HC | 0.110 |
| EG | 0.037 |
| LA | 0.094 |
| C_4_H_8_O_2_ | 0.138 |
| NaHCO_3_ | 0.067 |
| NaSO_4_ | 0.044 |

# *Figure S5 Cost diagram for SP1~SP7*

***Reference***

[1] L. Geng, T. Xiao, J. P. Jiang, K. F. Luo, Y. B. Yan, C. H. Liu, *Chemical Engineering Journal* **2024**, *496*.
